# Elevation of Extracellular Ca^2+^ Induces Store-Operated Calcium Entry via Calcium-Sensing Receptors: A Pathway Contributes to the Proliferation of Osteoblasts

**DOI:** 10.1371/journal.pone.0107217

**Published:** 2014-09-25

**Authors:** Fen Hu, Leiting Pan, Kai Zhang, Fulin Xing, Xinyu Wang, Imshik Lee, Xinzheng Zhang, Jingjun Xu

**Affiliations:** The Key Laboratory of Weak-Light Nonlinear Photonics, Ministry of Education, TEDA Applied Physics Institute and School of Physics, Nankai University, Tianjin, China; Faculdade de Medicina Dentária, Universidade do Porto, Portugal

## Abstract

**Aims:**

The local concentration of extracellular Ca^2+^ ([Ca^2+^]_o_) in bone microenvironment is accumulated during bone remodeling. In the present study we investigated whether elevating [Ca^2+^]_o_ induced store-operated calcium entry (SOCE) in primary rat calvarial osteoblasts and further examined the contribution of elevating [Ca^2+^]_o_ to osteoblastic proliferation.

**Methods:**

Cytosolic Ca^2+^ concentration ([Ca^2+^]_c_) of primary cultured rat osteoblasts was detected by fluorescence imaging using calcium-sensitive probe fura-2/AM. Osteoblastic proliferation was estimated by cell counting, MTS assay and ATP assay. Agonists and antagonists of calcium-sensing receptors (CaSR) as well as inhibitors of phospholipase C (PLC), SOCE and voltage-gated calcium (Cav) channels were applied to study the mechanism in detail.

**Results:**

Our data showed that elevating [Ca^2+^]_o_ evoked a sustained increase of [Ca^2+^]_c_ in a dose-dependent manner. This [Ca^2+^]_c_ increase was blocked by TMB-8 (Ca^2+^ release inhibitor), 2-APB and BTP-2 (both SOCE blockers), respectively, whereas not affected by Cav channels blockers nifedipine and verapamil. Furthermore, NPS2143 (a CaSR antagonist) or U73122 (a PLC inhibitor) strongly reduced the [Ca^2+^]_o_-induced [Ca^2+^]_c_ increase. The similar responses were observed when cells were stimulated with CaSR agonist spermine. These data indicated that elevating [Ca^2+^]_o_ resulted in SOCE depending on the activation of CaSR and PLC in osteoblasts. In addition, high [Ca^2+^]_o_ significantly promoted osteoblastic proliferation, which was notably reversed by BAPTA-AM (an intracellular calcium chelator), 2-APB, BTP-2, TMB-8, NPS2143 and U73122, respectively, but not affected by Cav channels antagonists.

**Conclusions:**

Elevating [Ca^2+^]_o_ induced SOCE by triggering the activation of CaSR and PLC. This process was involved in osteoblastic proliferation induced by high level of extracellular Ca^2+^ concentration.

## Introduction

Bone is constantly remodeling and maintaining homeostasis between formation and resorption. Reducing formation or increasing resorption may lead to bone loss, osteoporosis, eventually debilitating fractures [1]–[3]. Osteoblasts play a pivotal role in bone formation and mineralization by secreting bone matrix components and providing factors essential for osteoclast differentiation [4]–[6]. In the bone microenvironment, the resorptive action of osteoclasts results in a local increase of extracellular calcium concentration ([Ca^2+^]_o_) which can reach levels as high as 40 mM [7]. This high level of [Ca^2+^]_o_ has been suggested to regulate bone formation by stimulating osteoblastic proliferation, chemotaxis, differentiation and mineralization [8]–[10]. Indeed, *in vitro* studies showed that high [Ca^2+^]_o_ promoted proliferation in a number of osteoblast cell lines including rat calvarial osteoblasts [10].

In various cell types, the store operated calcium entry (SOCE) determines sustained cytosolic calcium concentration ([Ca^2+^]_c_) increase which is critical in regulating a variety of cellular functions including secretion, apoptosis, and more specifically proliferation [11]–[14]. SOCE is activated in response to a reduction of Ca^2+^ concentration in the intracellular endoplasmic reticulum (ER) stores. Under physiological conditions, receptor-mediated activation of the phospholipase C (PLC) induces the generation of inositol 1,4,5-trisphosphate (IP_3_) and subsequently triggers IP_3_ receptor-related Ca^2+^ release from ER, which may stimulate SOCE in turn [15]. The SOCE phenomenon was described in some osteoblast-like cells by previous studies [16]–[18]. Furthermore, it found that SOCE initiated by the stimulus of platelet-derived growth factor was involved in the proliferation of osteoblast-like MG-63 cells [18]. With respect to high [Ca^2+^]_o_-induced osteoblastic proliferation, the underlying intracellular signaling is largely unclear. Especially, it remains unknown whether the elevation of [Ca^2+^]_o_ can induce SOCE, and whether high [Ca^2+^]_o_-induced osteoblastic proliferation is conducted through SOCE in osteoblasts.

It was established that extracellular Ca^2+^ could activate the calcium-sensing receptors (CaSR), a member of G-protein coupled receptor family [19]. The activation of CaSR mediated intracellular Ca^2+^ release through PLC/IP_3_ pathway [19]–[21]. Functional expression of CaSR had been detected in different types of osteoblast-like cells including primary rat calvarial osteoblasts [22]–[28]. Studies so far suggested that CaSR was essential for osteoblast growth, differentiation and mineralization [23]–[27], therefore played a critical role in regulation of bone development and remodeling [28], [29]. However, the downstream signal pathway mediated by CaSR has not been extensively addressed. Interestingly, CaSR-induced Ca^2+^ release could trigger SOCE in breast cancer cells and cardiomyocytes [30], [31], whereas did not cause Ca^2+^ influx in renal collecting duct cells [32]. To our knowledge, whether CaSR activation can induce SOCE in osteoblasts is still unknown. In the present work, it was found that elevating [Ca^2+^]_o_ obviously induced a sustained rise of [Ca^2+^]_c_ in rat calvarial osteoblasts. Therefore, the aim of this study was to investigate the mechanism of [Ca^2+^]_c_ increase induced by [Ca^2+^]_o_ in rat calvarial osteoblasts. We asked whether the effects of [Ca^2+^]_o_ on [Ca^2+^]_c_ depended on the activation of CaSR-related PLC/IP_3_ signaling and SOCE. Furthermore, we examined the contribution of [Ca^2+^]_c_ increase to high [Ca^2+^]_o_-induced proliferation in primary rat calvarial osteoblasts.

## Materials and Methods

### Ethics Statement

The animal protocol in this study conformed to the Guide for the Care and Use of Laboratory Animals (*the Guide*, NRC 2011), and it was also approved by the Institutional Animal Care and Use Committee at Nankai University (Approval ID 201009080081).

### Animals and reagents

New born Wistar rats (3-day-old) were obtained from Academy of Military Medical Sciences (Tianjin, China). DMEM and fetal bovine serum (FBS) were from Gibco (USA) and HyClone (USA), respectively. Fura-2/AM was purchased from Biotium (USA). The rest of reagents, including trypsin, collagenase II, EGTA, DMSO, thapsigargin (TG), BAPTA-AM, TMB-8, 2-APB, BTP-2 (YM-58483), U73122, U73343, NPS2143, spermine, nifedipine and verapamil were purchased from Sigma-Aldrich (USA). CellTiter 96 AQueous One Solution Cell Proliferation Assay kit and CellTiter-Glo Luminescent Cell Viability Assay kit were purchased from Promega (USA).

### Osteoblasts isolation and culture

Rat calvarial osteoblasts were isolated and cultured as previously described [33], [34]. Briefly, anesthetized new born Wistar rats (3-day-old) were sacrificed by decapitation. Then, bone skulls were isolated from the soft tissue, and digested with collagenase. Calvarial cells were released by repeated digestion with trypsin. The isolated osteoblasts were cultured in DMEM medium containing 10% FBS at 37°C with 5% CO_2_.

### Measurement of cytosolic Ca^2+^ concentrations ([Ca^2+^]_c_)

Osteoblasts were loaded with 5 µM fura-2/AM in Hanks’ balanced salt solution (HBSS) (NaCl 150 mM, KCl 5.4 mM, CaCl_2_ 2 mM, MgCl_2_ 1 mM, glucose 10 mM and HEPES 10 mM, pH  = 7.4) for 1 h at room temperature. After washing extensively with HBSS, cells were bathed in fresh HBSS solution. [Ca^2+^]_c_ was measured with calcium imaging system built on an inverted fluorescence microscope (Olympus IX51). The Ca^2+^ indicator fura-2 was alternately excited at 340 nm and 380 nm with a Lambda 10–2 sutter. Fluorescence images (filtered at 515 nm±25 nm) were captured by a CCD camera (CoolSNAP fx-M) and quantitated with MetaFluor. [Ca^2+^]_c_ was represented by the ratio of fluorescence intensity at 340 nm/fluorescence intensity at 380 nm (F340/F380). At least three independent experiments were done for each condition. One curve of calcium changes was plotted as the representation of other similar traces. Ca^2+^-free HBSS solution was made by substituting MgCl_2_ for CaCl_2_ at the same concentration.

### Proliferation Assay

The proliferation of osteoblasts was assessed by morphological observations and direct cell counting. The number of viable cells in proliferation was further determined by MTS assay (CellTiter 96 AQueous One Solution Cell Proliferation Assay kit) and ATP assay (CellTiter-Glo Luminescent Cell Viability Assay kit), respectively. For morphological observations, osteoblasts were plated in 35 mm culture dishes (∼5×10^4^ cells/dish) with DMEM containing 5% FBS at 37°C. Then, the pretreated-cells in each dish were monitored by an inverted light microscope (Olympus IX51) at 0, 24, 48 and 72 h in turn. In the meantime, the cell numbers in each dish were measured from at least five regions (1 mm×1 mm grids) at the indicated time. For MTS and ATP assays, osteoblasts were seeded into 96-well plate at ∼1×10^4^ cells/well at 37°C in DMEM with 5% FBS and incubated overnight before treating with or without test agents for 72 h. The MTS assay was performed by directly adding 20 µl of the AQueous One Solution Reagent to culture wells (100 µl/well), incubating for 4 h and then recording the absorbance at 490 nm (A_490_) with an ELISA reader (Bio-Rad Imark Microplate Reader). The ATP assay was carried out by adding 100 µl of the CellTiter-Glo Reagent (Buffer plus Substrate) to each well, then mixing contents for 2 minutes on an orbital shaker to induce cell lysis. After that the plate was incubated for 10 minutes to stabilize luminescent signal. The luminescent signal was measured by a luminometer (GloMax Multi Jr Detection System, Promega, USA). The ATP concentration in each well was derived from the standard curve.

### Statistical analysis

All data passed the normality test and were presented as mean ± standard deviation. The statistical comparison between two groups was carried out using Student’s t-test (Origin 8.0), and the analysis for multiple groups was using Dunnett’s test (SPSS 18.0, one-way ANOVA). *P*<0.05 was considered to be statistically significant. The values of half maximal effective concentration (EC_50_) were calculated according to the dose-response curve fitting with the logistic equation: 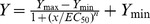, where *Y* is the response, *Y_max_* is the asymptotic maximum, *Y_min_* is the asymptotic minimum, *x* is the extracellular calcium concentration and *n* is the Hill coefficient.

## Results

### Thapsigargin induced SOCE in rat calvarial osteoblasts

**Figure 1:**
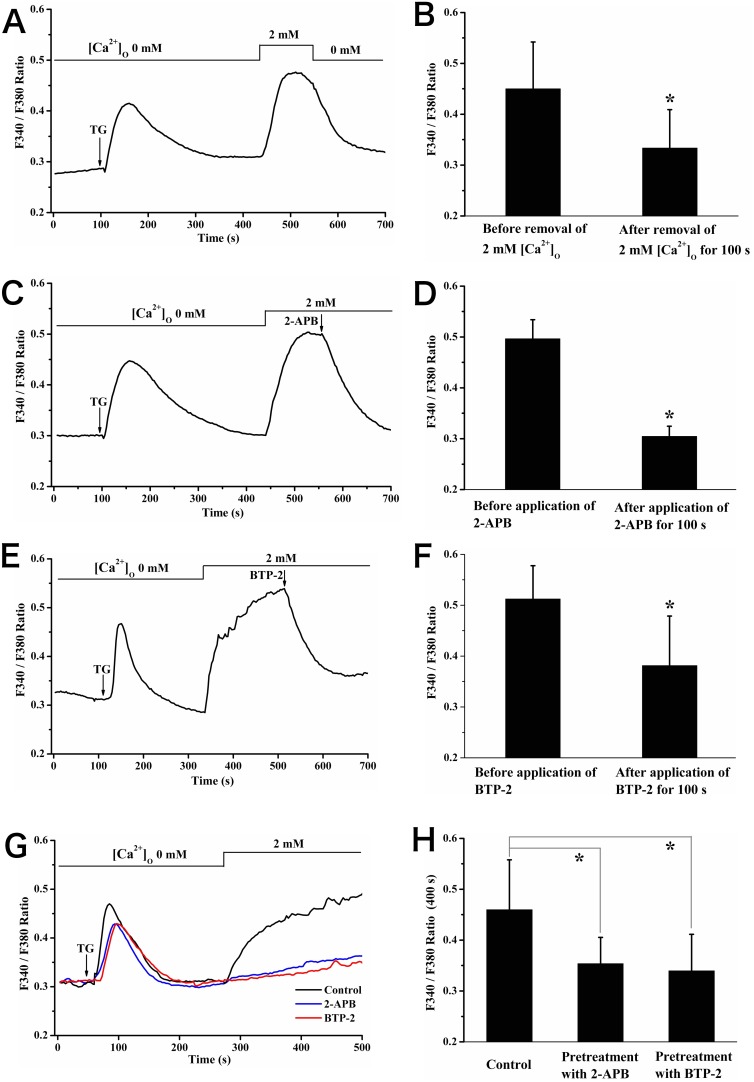
TG induced SOCE in rat calvarial osteoblasts. (A) After calcium store depletion by a calcium pump blocker TG (1 µM) in Ca^2+^-free buffer, addition of 2 mM external Ca^2+^ resulted in obvious calcium entry; then, further removal of external Ca^2+^ caused [Ca^2+^]_c_ decrease to baseline, suggesting the putative response for SOCE. (C, E) [Ca^2+^]_c_ increase was caused by TG (1 µM) in Ca^2+^-free HBSS, followed by application of 25 µM 2-APB or 20 µM BTP-2 during the high [Ca^2+^]_c_ plateau induced by re-addition of 2 mM external Ca^2+^, resulting in return to baseline [Ca^2+^]_c_. Statistic data of ratio of F340/F380 before and after the application of Ca^2+^ free HBSS (B), 2-APB (D) and BTP-2 (F). * showed *P*<0.05. (G) 1 µM TG was added after pretreatment with 25 µM 2-APB or 20 µM BTP-2 for 15 min, then, further addition of 2 mM external Ca^2+^ had no effect on [Ca^2+^]_c_ change. (H) Summary of the ratio of F340/F380 at 400 s from experiments shown in (G), * showed *P*<0.05.

Firstly, we checked the ability of generating SOCE in rat calvarial osteoblasts with ER Ca^2+^-pump blocker thapsigargin (TG), a drug widely used to test SOCE. It was seen from Figure 1A that the application of TG (1 µM) evoked a transient [Ca^2+^]_c_ rise mediated by Ca^2+^ release from Ca^2+^ stores with nominally Ca^2+^-free HBSS. Adding 2 mM CaCl_2_ after [Ca^2+^]_c_ returning to the basal level triggered [Ca^2+^]_c_ increase due to Ca^2+^ entry. This Ca^2+^ entry was strongly inhibited by the application of potent SOCE blockers 2-APB (25 µM) [35], [36] (value of F340/F380: 0.50±0.04 before application of 2-APB *vs*. 0.30±0.02 after application of 2-APB for 100 s, *P*<0.05) or BTP-2 (YM-58483, 20 µM) [37] (value of F340/F380: 0.51±0.07 before application of BTP-2 *vs*. 0.38±0.10 after application of BTP-2 for 100 s, *P*<0.05) during the high [Ca^2+^]_c_ plateau evoked by adding 2 mM CaCl_2_ (Figure 1C–F). In addition, Ca^2+^ entry was evidently abolished when cells were pretreatment with 2-APB (25 µM) or BTP-2 (20 µM) before adding TG (value of F340/F380 at 400 s: 0.46±0.10 for control *vs*. 0.35±0.05 for 2-APB *vs*. 0.34±0.07 for BTP-2, *P*<0.05; Figure 1G and H). Taken together, these data confirmed the existence of SOCE in rat calvarial osteoblasts and the efficient inhibition of 2-APB and BTP-2 on SOCE.

### Elevating [Ca^2+^]_o_ induced increases in [Ca^2+^]_c_ in rat calvarial osteoblasts

**Figure 2:**
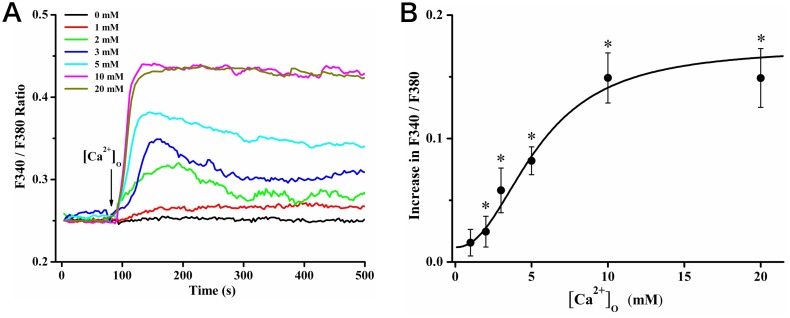
Elevated [Ca^2+^]_o_ resulted in [Ca^2+^]_c_ increases in rat calvarial osteoblasts. (A) Representative tracings of [Ca^2+^]_c_ responses induced by [Ca^2+^]_o_ at 0, 1, 2, 3, 5, 10 and 20 mM, respectively. (B) The statistic peak values of increase in F340/F380 ratio were plotted against [Ca^2+^]_o_ (n = 15 for each case), * showed *P*<0.05. The smooth curve represented the fitting to the equation of 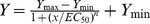 with an EC_50_ value of 5.4±1.2 mM and Hill coefficient (n) of 2.2

The effects of elevating [Ca^2+^]_o_ on [Ca^2+^]_c_ were measured by calcium imaging. A rise in F340/F380 ratio indicated an increase in [Ca^2+^]_c_. Representative [Ca^2+^]_c_ profiles were shown in Figure 2A. Elevating [Ca^2+^]_o_ from 0 mM to 1, 2, 3, 5, 10 and 20 mM resulted in a rapid increase in [Ca^2+^]_c_ followed by a sustained high [Ca^2+^]_c_ plateau. The increase of [Ca^2+^]_c_ was dependent on the level of [Ca^2+^]_o_. We measured the peak value of [Ca^2+^]_c_ increase and plotted it against the concentrations of extracellular Ca^2+^ (Figure 2B). The dose-dependent curve was fitted with an EC_50_ of 5.4±1.2 mM.

### Voltage-gated calcium channels did not contribute to [Ca^2+^]_o_-induced [Ca^2+^]_c_ increase

**Figure 3:**
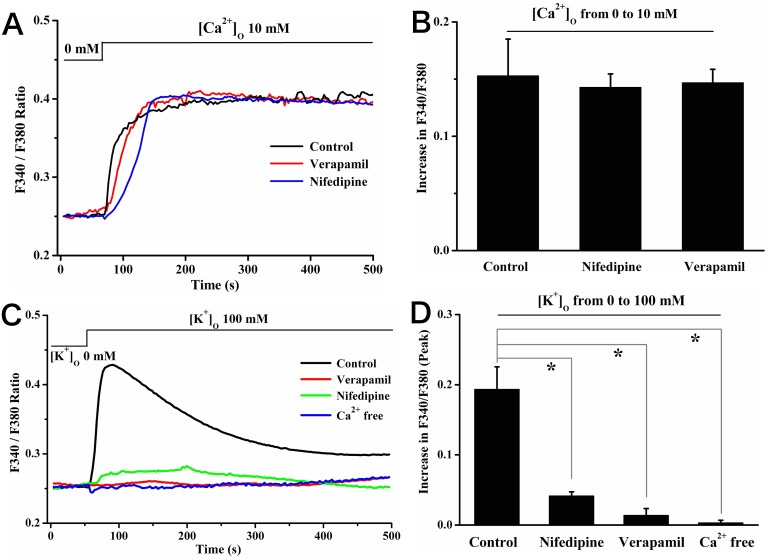
Nifedipine or verapamil had no effect on [Ca^2+^]_o_-induced [Ca^2+^]_c_ increase in rat calvarial osteoblasts. (A) Representative tracings of [Ca^2+^]_c_ changes caused by elevating [Ca^2+^]_o_ (10 mM) alone (control) and in the presence of nifedipine (10 µM) or verapamil (10 µM). Nifedipine or verapamil was added for 15 min before elevating [Ca^2+^]_o_. (B) Summary of the changes in F340/F380 at 250 s after the elevation of [Ca^2+^]_o_ from experiments shown in (A). (C) Typical tracings of [Ca^2+^]_c_ changes caused by elevating [K^+^]_o_ (100 mM) alone (control) and in the presence of Ca^2+^ free HBSS, nifedipine (10 µM) or verapamil (10 µM). Nifedipine or verapamil was added for 15 min before elevating [K^+^]_o_. (D) Summary of the peak values of increase in F340/F380 after the elevation of [K^+^]_o_ from experiments shown in (C).

Because rat calvarial osteoblasts expressed voltage-gated calcium (Cav) channels [38], [39], we tested whether Cav channels contributed to [Ca^2+^]_o_-induced [Ca^2+^]_c_ increase. It found that pretreatment the cells with Cav blockers nifedipine (10 µM) [27], [40] or verapamil (10 µM) [40] had little influence on the [Ca^2+^]_c_ increase evoked by elevating [Ca^2+^]_o_ (10 mM) as show in Figure 3A. The peak values for [Ca^2+^]_c_ increase were not different from that of control (value of increase in F340/F380 at 250 s: 0.15±0.03 for control *vs*. 0.14±0.01 for nifedipine *vs*. 0.15±0.01 for verapamil, *P*>0.05; Figure 3B). To verify the effectiveness of these two Cav blockers, a high [K^+^]_o_ experiment was performed as positive control. Data showed that elevating [K^+^]_o_ from 0 mM to 100 mM triggered a rapid increase of [Ca^2+^]_c_ (black line, Figure 3C), which was known to be attributed to Ca^2+^ entry through Cav channels (blue line, Figure 3C). Meanwhile, both verapamil and nifedipine at the used concentrations could block this [K^+^]_o_-induced Ca^2+^ entry (peak value of increase in F340/F380: 0.19±0.03 for control *vs*. 0.042±0.006 for nifedipine *vs*. 0.014±0.009 for verapamil, *P*<0.05; Figure 3C and D). These data together indicated that Cav channels did not participate in the process of [Ca^2+^]_o_-induced [Ca^2+^]_c_ increase.

### SOCE played key roles in the [Ca^2+^]_c_ increase induced by elevating [Ca^2+^]_o_

**Figure 4:**
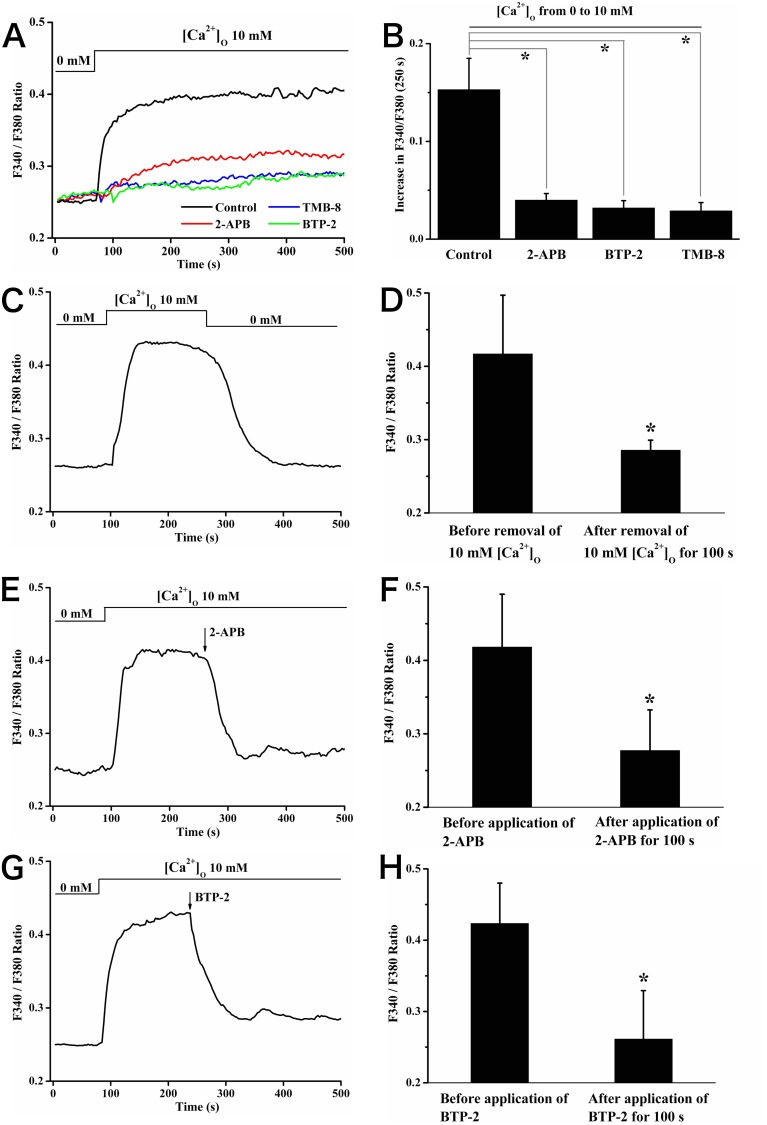
[Ca^2+^]_o_-induced [Ca^2+^]_c_ increase was blocked by 2-APB, BTP-2 and TMB-8 in rat calvarial osteoblasts, respectively. (A) Typical tracings of [Ca^2+^]_c_ responses resulted from elevating [Ca^2+^]_o_ (10 mM) in the absence (control) and in the presence of 2-APB (25 µM), BTP-2 (20 µM), or TMB-8 (50 µM). Such reagents were added for 15 min before the elevation of [Ca^2+^]_o_. (B) Summary of the changes in F340/F380 at 250 s after the elevation of [Ca^2+^]_o_ from experiments shown in (A), * showed *P*<0.05 comparing with control. (C, E, G) Representative tracings showing the effects of application of Ca^2+^ free HBSS, 25 µM 2-APB or 20 µM BTP-2 on the high [Ca^2+^]_c_ plateau induced by elevating [Ca^2+^]_o_. Statistic data of the ratio of F340/F380 before and after the application of Ca^2+^ free HBSS (D), 2-APB (F) and BTP-2 (H), * showed *P*<0.05.

To investigate whether SOCE was associate with [Ca^2+^]_o_-induced [Ca^2+^]_c_ increase, a series of experiments were carried out. Firstly, the rise of [Ca^2+^]_c_ induced by 10 mM [Ca^2+^]_o_ was decreased significantly (value of increase in F340/F380 at 250 s: 0.15±0.03 for control vs. 0.03±0.01 for TMB-8 *vs*. 0.04±0.01 for 2-APB *vs*. 0.03±0.01 for BTP-2, *P*<0.05; Figure 4A and B) when cells were pretreated with 50 µM TMB-8 (a calcium release inhibitor) [41], 25 µM 2-APB and 20 µM BTP-2, respectively. Secondly, substitution with Ca^2+^ free HBSS during the [Ca^2+^]_o_-induced high [Ca^2+^]_c_ plateau rapidly reduced [Ca^2+^]_c_ to the baseline, indicating the sustained high [Ca^2+^]_c_ plateau was attributed to Ca^2+^ entry (value of F340/F380: 0.42±0.08 before removal of 10 mM [Ca^2+^]_o_ *vs*. 0.29±0.01 after removal of 10 mM [Ca^2+^]_o_ for 100 s, *P*<0.05; Figure 4C and D). Similar responses were observed after the application of 25 µM 2-APB (value of F340/F380: 0.42±0.07 before application of 2-APB *vs*. 0.28±0.06 after application of 2-APB for 100 s, *P*<0.05) or 20 µM BTP-2 (value of F340/F380: 0.42±0.06 before application of BTP-2 vs. 0.26±0.07 after application of BTP-2 for 100 s, *P*<0.05) (Figure 4E–H). Taken together, these results above revealed that elevating [Ca^2+^]_o_ induced the activation of SOCE underlying the increase of [Ca^2+^]_c_.

### [Ca^2+^]_o_-induced SOCE was dependent on the activation of CaSR

**Figure 5:**
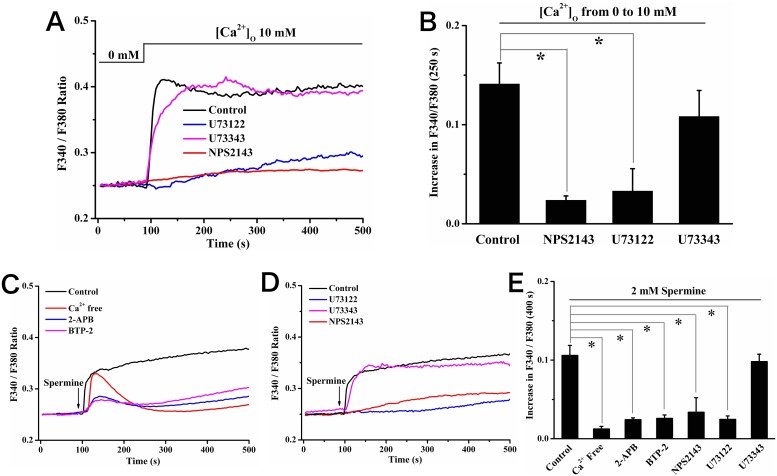
[Ca^2+^]_o_-induced [Ca^2+^]_c_ increase was dependent on the activation of CaSR/PLC signaling in rat calvarial osteoblasts. (A) Representative tracings of [Ca^2+^]_c_ changes induced by elevating [Ca^2+^]_o_ (10 mM) alone (control) and in the presence of NPS2143 (10 µM), U73122 (5 µM) or U73343 (5 µM). Such reagents were added 15 min before application of the elevation of [Ca^2+^]_o_. (B) Summary of the changes in F340/F380 at 250 s after the elevation of [Ca^2+^]_o_ from experiments shown in (A), * showed *P*<0.05, compared with control in each group. (C) Typical tracings of [Ca^2+^]_c_ responses induced by induced by 2 mM spermine in the presence (black) and absence (red) of external Ca^2+^. Cells were pretreated with 25 µM 2-APB (blue) or 20 µM BTP-2 (purple) for 15 min prior to spermine (2 mM) in Ca^2+^-containing HBSS. (D) Representative tracings of [Ca^2+^]_c_ changes in response to 2 mM spermine in the presence of NPS2143 (10 µM), U73122 (5 µM) or U73343 (5 µM) in Ca^2+^-containging HBSS. Such reagents were added 15 min before adding spermine. (E) Summary of the changes in F340/F380 at 400 s after the stimulation with spermine in the presence Ca^2+^ free HBSS, 2-APB, BTP-2, NPS2143, U73122 or U73343 from experiments shown in C and D, * showed *P*<0.05 comparing with control (spermine alone) in each group.

The role of CaSR-PLC/IP_3_ signaling in [Ca^2+^]_o_-induced SOCE was examined in the following experiments. Firstly, the elevating [Ca^2+^]_o_-induced [Ca^2+^]_c_ increase was almost abolished when cells were pretreated with a specific CaSR antagonist NPS2143 (10 µM) [27] (value of increase in F340/F380 at 250 s: 0.14±0.02 for control *vs*. 0.024±0.004 for NPS2143, *P*<0.05; Figure 5A and B), suggesting the contribution of CaSR to SOCE. Moreover, U73122 (5 µM) [36], [42], a potent PLC inhibitor, attenuated the [Ca^2+^]_c_ rise significantly (value of increase in F340/F380 at 250 s: 0.14±0.02 for control *vs*. 0.03±0.02 for U73122, *P*<0.05; Figure 5A and B), indicating the involvement of PLC. In addition, we tested the effects of spermine, a polycationic agonist of CaSR, taking it as a positive control. It can be seen from Figure 5C–E, spermine (2 mM) triggered [Ca^2+^]_c_ increase with similar characteristics to that of [Ca^2+^]_c_ change resulted from elevated [Ca^2+^]_o_. As expected, the removal of extracellular calcium or pretreatment with 2-APB (25 µM) and BTP-2 (20 µM) suppressed the sustained [Ca^2+^]_c_ increase induced by spermine in Ca^2+^-containing HBSS (Figure 5C and E). It also failed to evoke a [Ca^2+^]_c_ increase by spermine in the presence of NPS2143 (10 µM) or U73122 (5 µM) (Figure 5D and E) in Ca^2+^-containing buffer. In contrast, U73343, an inactive analog of U73122, had little effect on the [Ca^2+^]_c_ increase induced by either [Ca^2+^]_o_ (value of increase in F340/F380 at 250 s: 0.14±0.02 for control *vs*. 0.11±0.03 for U73343, *P*>0.05; Figure 4A and B) or spermine (value of increase in F340/F380 at 400 s: 0.11±0.01 for control *vs*. 0.10±0.01 for U73343, *P*>0.05; Figure 5D and E). Taken together, these data suggested an essential role for CaSR activation and the subsequent PLC-IP_3_ pathway in [Ca^2+^]_o_ elevation-induced SOCE.

### SOCE was involved in the high [Ca^2+^]_o_-induced proliferation

**Figure 6:**
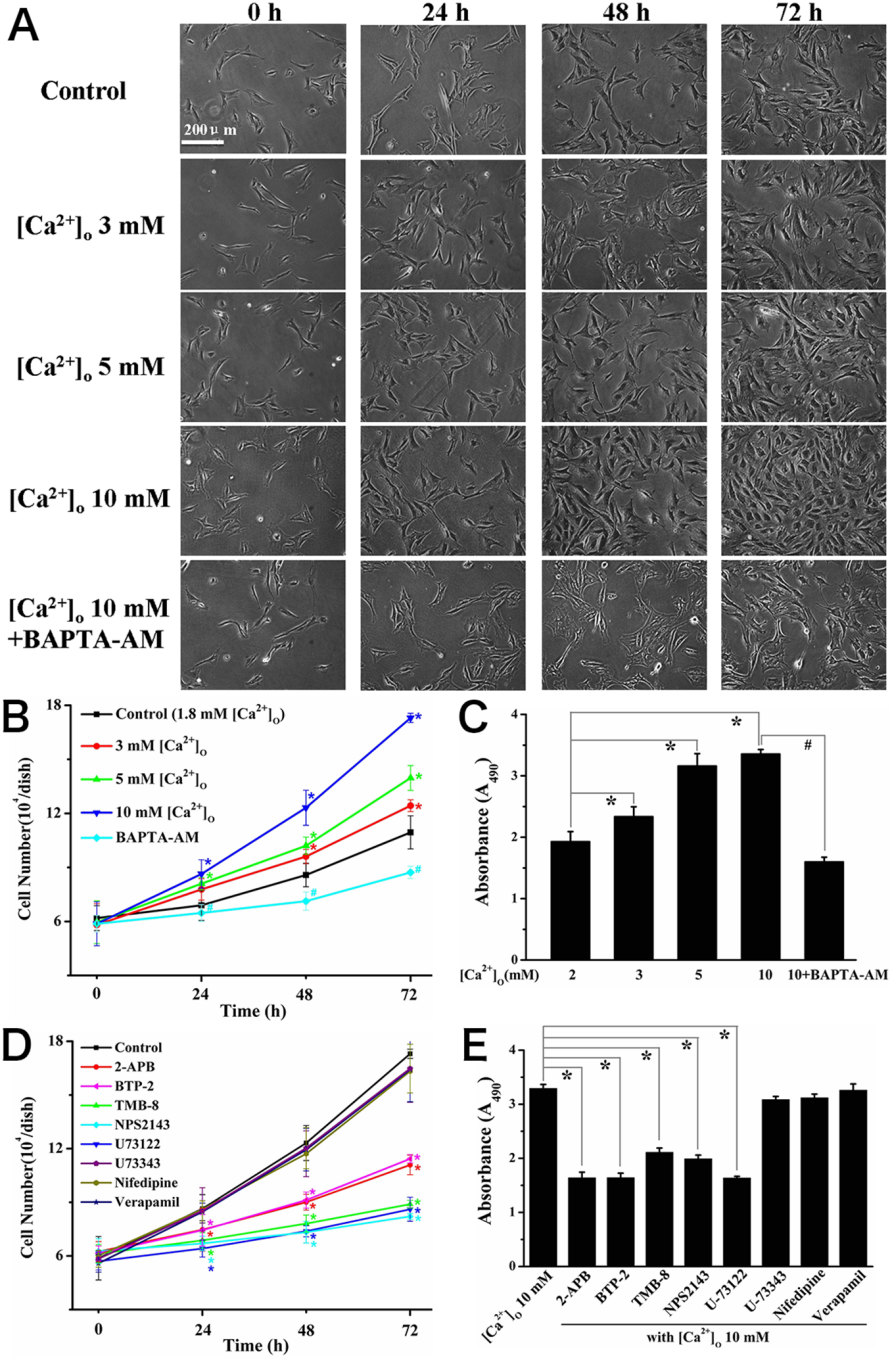
[Ca^2+^]_o_-induced SOCE was involved in the high [Ca^2+^]_o_-induced proliferation in rat calvarial osteoblasts. (A) Osteoblasts were cultured in medium containing different levels of [Ca^2+^]_o_ or in a medium with 2 µM BAPTA-AM+10 mM [Ca^2+^]_o_. Typical cell morphological images were captured at 0 h, 24 h and 48 h and 72 h using a 10× objective. (B) Statistic data of cell numbers from experiments shown in (A). Each group of cells were grown in triplicate dishes and counted at different time points by measuring at least five regions (1 mm×1 mm grids). *P<0.05, compared with control group (normal DMEM medium); # *P*<0.05, compared with 10 mM [Ca^2+^]_o_ group. (C) Summary of absorbance (A_490_) in each group. Absorbance (A_490_) assessed by MTS assay is proportional to the number of living cells. Osteoblasts were incubated for 72 h in culturing medium with different levels of [Ca^2+^]_o_ or in a medium with 2 µM BAPTA-AM+10 mM [Ca^2+^]_o_ (n = 7 for each case), * showed *P*<0.05, compared with [Ca^2+^]_o_  = 1.8 mM group; # showed *P*<0.05, compared with [Ca^2+^]_o_  = 10 mM group. (D) Statistic data of cell numbers in each group at different time points. Osteoblasts were cultured in medium containing 10 mM [Ca^2+^]_o_ alone or together with 2-APB (25 µM), BTP-2 (20 µM), TMB-8 (50 µM), NPS2143 (10 µM), U73122 (5 µM), U73343 (5 µM), nifedipine (10 µM) and verapamil (10 µM), respectively. * showed *P*<0.05 in comparison with [Ca^2+^]_o_  = 10 mM group. (E) Summary of absorbance (A_490_) measured after culturing for 72 h in [Ca^2+^]_o_ = 10 mM medium alone or 2-APB (25 µM), BTP-2 (20 µM), TMB-8 (50 µM), NPS2143 (10 µM), U73122 (5 µM), U73343 (5 µM), nifedipine (10 µM) and verapamil (10 µM) (n = 7 for each case), respectively. * showed *P*<0.05 compared with [Ca^2+^]_o_ = 10 mM group.

To investigate the effects of [Ca^2+^]_o_ on the proliferation capacity of osteoblasts and the contribution of [Ca^2+^]_o_-induced [Ca^2+^]_c_ increase to proliferation of osteoblasts, we carried out a series of experiments to assess the cell proliferation in different levels of [Ca^2+^]_o_ with or without various inhibitors by morphological observations and cell counting, as well as estimating the proliferating activity by MTS and ATP assays. Morphological images of osteoblasts were obtained with medium containing 1.8, 3, 5, 10 mM [Ca^2+^]_o_ at 0, 24, 48 and 72 h, respectively. It was found that the cell numbers in medium with higher [Ca^2+^]_o_ were increased significantly in comparison with control (normal DMEM medium, [Ca^2+^]_o_  = 1.8 mM) in a concentration-dependent and time-dependent manner (Figure 6A and B). Meanwhile, the absorbance (A_490_) (MTS assay) (Figure 6C) and ATP concentration (ATP assay) (Figure S1) were increased with higher [Ca^2+^]_o_ medium at 72 h. In addition, this increase of proliferation induced by 10 mM [Ca^2+^]_o_ was completely blocked by an intracellular calcium chelator BAPTA-AM (2 µM) (Figure 6A–C and Figure S1). Furthermore, 10 mM [Ca^2+^]_o_-stimulated cell proliferation was decreased significantly in the presence of 2-APB (25 µM), BTP-2 (20 µM), TMB-8 (50 µM), NPS2143 (10 µM) and U73122 (5 µM), respectively, while treating osteoblasts with U73343 (5 µM), nifedipine (10 µM) or verapamil (10 µM) had little influence on cell proliferation (Figure 6D and E, Figure S1 and S2). These data indicated that the CaSR activation-induced SOCE participated in the process of high [Ca^2+^]_o_-promoted cell proliferation in rat calvarial osteoblasts.

## Discussion

In the present study, we found that elevating [Ca^2+^]_o_ triggered [Ca^2+^]_c_ increases in a dose-dependent manner with a EC_50_ of 5.4±1.2 mM in rat calvarial osteoblasts. This [Ca^2+^]_c_ increase was abolished by SOCE blockers 2-APB and BTP-2, or Ca^2+^ release inhibitor TMB-8, while not affected by Cav channels antagonists nifedipine or verapamil. Furthermore, specific CaSR antagonist NPS2143 or PLC inhibitor U73122 strongly reduced the [Ca^2+^]_c_ increase resulted from elevating [Ca^2+^]_o_. These data indicated that elevating [Ca^2+^]_o_ induced SOCE in osteoblasts, which was dependent on the activation of CaSR and PLC. The SOCE route, rather than Cav channels contributed to elevation [Ca^2+^]_o_-induced [Ca^2+^]_c_ increase. In addition, high levels of [Ca^2+^]_o_ promoted the proliferation of osteoblasts. This promotion was also significantly inhibited by 2-APB, BTP-2, TMB-8, NPS2143 and U73122, respectively, suggesting a contribution of CaSR-related PLC/IP_3_-SOCE pathway to high [Ca^2+^]_o_-induced proliferation in osteoblasts.

The local concentration of extracellular Ca^2+^ in bone microenvironment is fluctuant during bone remodeling, and it regulates the functions of osteoblasts including proliferation. However, the intracellular signaling activated by extracellular Ca^2+^ in osteoblasts is largely unknown. The most significant finding of this study was that we found elevation of extracellular Ca^2+^ concentration induced SOCE by triggering the activation of CaSR and PLC. Firstly, we demonstrated that elevated [Ca^2+^]_o_ triggered a sustained [Ca^2+^]_c_ increase (Figure 2A). This [Ca^2+^]_c_ increase played key roles in cell proliferation (Figure 6B; experiment with BAPTA-AM). We further showed that extracellular Ca^2+^ entry contributed mostly to the sustained [Ca^2+^]_c_ increase (Figure 4C). In most cell types, SOCE mediated extracellular Ca^2+^ entry for ER Ca^2+^ store refilling [11], and more importantly, the SOCE phenomenon was found in several osteoblastic cell lines including rat calvarial osteoblasts [16]–[18]. On the other hand, some papers reported the functional expression of Cav channels in osteoblasts, which regulated the cell proliferation and differentiation dependent on the type and expression level of Cav channels [38], [39]. Thus, Cav channels may also serve as candidate channels accounting for Ca^2+^ capacitive entry. We addressed these two presumptions in this study. Our data showed that SOCE blockers 2-APB, BTP-2, or Ca^2+^ release inhibitor TMB-8 almost abolished the [Ca^2+^]_o_-triggered [Ca^2+^]_c_ increase (Figure 4), whereas Cav channels antagonists had little effects (Figure 3). These results suggested that [Ca^2+^]_o_-induced [Ca^2+^]_c_ increase was most likely through the activation of SOCE route rather than Cav channels. With respect to the molecular components of SOCE conducting Ca^2+^ entry, some transient receptor potential (TRP) channels especially TRPC subfamily were considered for candidate SOCE machinery in osteoblasts as well as other cell types. Many members of TRP channels had been identified to exist in osteoblasts and served as SOCE machinery [43]–[45]. For instance, reports showed that the TRPC1 channel played a key role in SOCE and cell proliferation induced by platelet-derived growth factor in osteoblast-like MG-63 cell line [18], while TRPC3 was related to vitamin D-induced SOCE in chick skeletal muscle [46] and ROS 17/2.8 rat osteoblastic cells [47]. However, which member of TRPC channels actually contributed to elevated [Ca^2+^]_o_-triggered [Ca^2+^]_i_ increases needs further investigation.

In terms of how extracellular Ca^2+^ activated SOCE, it was known that extracellular Ca^2+^ could stimulate G protein-PLC pathway by activating CaSR in various cell types. Then the following production of IP_3_ caused Ca^2+^ release. Functional CaSR was expressed in different types of osteoblast-like cells including primary rat calvarial osteoblasts [22]–[28]. However, it is not clear whether CaSR activation can cause SOCE in osteoblasts. In the present study, we found that elevated [Ca^2+^]_o_-induced [Ca^2+^]_c_ increase was strongly blocked by specific CaSR antagonist NPS2143 and PLC inhibitor U73122. Similar responses were observed when the cells were stimulated with spermine, a specific CaSR agonist (Figure 5). Furthermore, the EC_50_ value for [Ca^2+^]_o_-induced [Ca^2+^]_c_ increase was 5.4 mM, which was close to the EC_50_ value of CaSR in response to extracelluar Ca^2+^ [19]. These data together indicated that extracellular Ca^2+^-induced SOCE was dependent on the activation of CaSR-related PLC/IP_3_ pathway.

Another interesting finding of the present study was that we demonstrated the contribution of SOCE and CaSR-related PLC/IP_3_ signaling in high [Ca^2+^]_o_-induced proliferation in primary rat calvarial osteoblasts. It has been established that high level of [Ca^2+^]_o_ promoted the proliferation in a number of osteoblastic cell lines. A few papers reported that multiple intracellular signal pathways such as calcium/calmodulin, MEK/ERK could be activated by high [Ca^2+^]_o_ stimulation and played roles in mediating high [Ca^2+^]_o_-promoted proliferation in different types of osteoblasts [9], [10], [48]–[51]. But how activation of these signal pathways leads to osteoblastic proliferation is still unclear now. As reported, high [Ca^2+^]_o_ activated PLC, probably through a G-protein-coupled receptor mechanism, which then caused accumulation of IP_3_ and mobilization of intracellular calcium, subsequently activated calcium/calmodulin/CaMKII signaling [9]. Furthermore, in primary human osteoblasts and MG-63 cells, high [Ca^2+^]_o_-stimulated proliferation was dependent on sustained activation of extracellular signal-regulated kinase 1 (ERK1) and ERK2, but other mitogen-activated protein (MAP) kinase signal pathways, p38 MAP kinase and SAPK/JNK, were not activated by [Ca^2+^]_o_ in osteoblasts [10]. ERK pathway was also found to participate in mediating bone morphogenetic protein (BMP)-2 gene expression induced by elevated [Ca^2+^]_o_ in human dental pulp cells [48]. High [Ca^2+^]_o_ was required for phosphate-dependent ERK1/2 phosphorylation and regulation of mineralization-associated genes in osteoblasts [49]. Besides, other signal pathways including calcineurin/NFAT and cAMP/PKA were involved in other cell functions such as gene expression induced by high extracellular calcium [50], [51]. In addition to the above mechanisms, the route of SOCE also played an important role in proliferation by inducing sustained [Ca^2+^]_c_ increase in various cell types including embryonic stem cells and smooth muscle cells [12]–[14]. Therefore, it’s necessary to concern about its role in the high [Ca^2+^]_o_-induced proliferation of osteoblasts. In this study, our data showed that intracellular calcium chelator BAPTA-AM reversed the high [Ca^2+^]_o_-induced proliferation. 2-APB, BTP-2, TMB-8, NPS2143 or U73122 also reduced the [Ca^2+^]_o_-induced proliferation (Figure 6), indicating the contribution of CaSR/PLC activation-evoked SOCE to high [Ca^2+^]_o_-induced proliferation of osteoblasts. In contrast, nifedipine or verapamil had little influence on the proliferation, suggesting that Cav channels were not involved. These results added a new insight to the current understanding on the role of extracellular calcium in osteoblasts proliferation.

In summary, we demonstrated that the elevation of [Ca^2+^]_o_ could stimulate CaSR, activate PLC, then trigger SOCE and consequently result in a sustained increase of [Ca^2+^]_c_. This process was involved in osteoblastic proliferation induced by high level of extracellular Ca^2+^ concentration. These findings may lead to new insights in the mechanisms of osteoblastic proliferation, and could provide some cellular basis for physiological regulation of bone remodeling.

## Supporting Information

Figure S1 **10 mM [Ca^2+^]_o_-induced increase in ATP concentration was blocked by inhibitors including BAPTA-AM, 2-APB, BTP-2, TMB-8, NPS2143 and U73122, but not affected by U73122 inactive analog U73343, voltage-gated calcium channels blockers nifedipine and verapamil in rat calvarial osteoblasts.** (A) Statistic data of ATP concentration in each group. The quantitation of the ATP concentration assessed by ATP assay is proportional to the number of viable cells present in culture. Osteoblasts were incubated for 72 h in culturing medium with different levels of [Ca^2+^]_o_ or in a medium with 2 µM BAPTA-AM+10 mM [Ca^2+^]_o_ (n = 7 for each case), * showed *P*<0.05, compared with [Ca^2+^]_o_  = 1.8 mM group; # showed *P*<0.05, compared with [Ca^2+^]_o_  = 10 mM group. (B) Statistic data of ATP concentration measured after culturing for 72 h in [Ca^2+^]_o_ = 10 mM medium alone or together with 2-APB (25 µM), BTP-2 (20 µM), TMB-8 (50 µM), NPS2143 (10 µM), U73122 (5 µM), U73343 (5 µM), nifedipine (10 µM) and verapamil (10 µM) (n = 5 for each case), respectively. * showed *P*<0.05 in comparison with [Ca^2+^]_o_  = 10 mM group.
(TIF)

Figure S2 **High [Ca^2+^]_o_-induced increase in cell numbers was blocked by inhibitors including 2-APB, BTP-2, TMB-8, NPS2143 and U73122, respectively, but not affected by voltage-gated calcium channels blockers nifedipine and verapamil in rat calvarial osteoblasts.** Osteoblasts were cultured in medium with 10 mM [Ca^2+^]_o_ alone or together with 2-APB (25 µM), BTP-2 (20 µM), TMB-8 (50 µM), NPS2143 (10 µM), U73122 (5 µM), U73343 (5 µM), nifedipine (10 µM) and verapamil (10 µM). Representative cell morphological images were captured at 0 h, 24 h, 48 h and 72 h using a 10× objective.
(TIF)
