# Supplementary figures and images for: Elevation of Extracellular Ca2+ Induces Store-Operated Calcium Entry via Calcium-Sensing Receptors: A Pathway Contributes to the Proliferation of Osteoblasts
Source: PLoS One. 2014 Sep 25;9(9):e107217. doi: 10.1371/journal.pone.0107217 (PMC4177836; doi:10.1371/journal.pone.0107217)

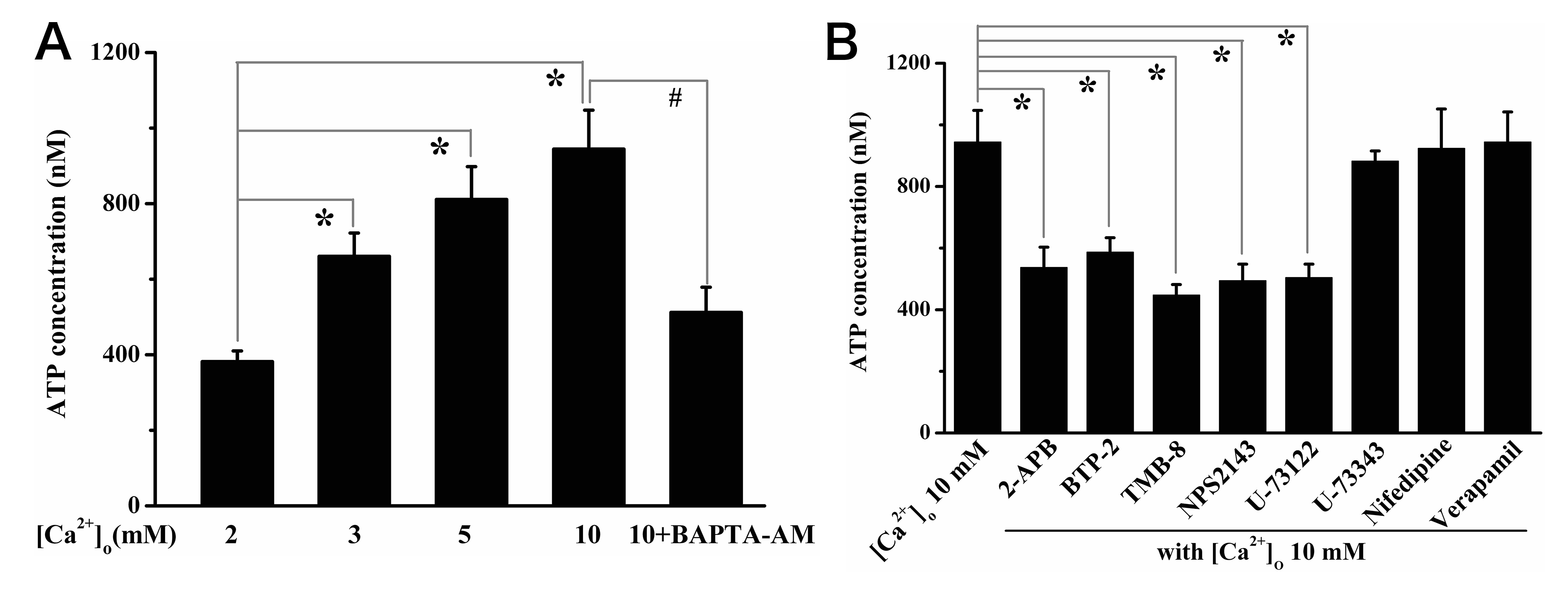

Supplement: Figure S1 — 10 mM [Ca2+]o-induced increase in ATP concentration was blocked by inhibitors including BAPTA-AM, 2-APB, BTP-2, TMB-8, NPS2143 and U73122, but not affected by U73122 inactive analog U73343, voltage-gated calcium channels blockers nifedipine and verapamil in rat calvarial osteoblasts. (A) Statistic data of ATP concentration in each group. The quantitation of the ATP concentration assessed by ATP assay is proportional to the number of viable cells present in culture. Osteoblasts were incubated for 72 h in culturing medium with different levels of [Ca2+]o or in a medium with 2 µM BAPTA-AM+10 mM [Ca2+]o (n = 7 for each case), * showed P<0.05, compared with [Ca2+]o = 1.8 mM group; # showed P<0.05, compared with [Ca2+]o = 10 mM group. (B) Statistic data of ATP concentration measured after culturing for 72 h in [Ca2+]o = 10 mM medium alone or together with 2-APB (25 µM), BTP-2 (20 µM), TMB-8 (50 µM), NPS2143 (10 µM), U73122 (5 µM), U73343 (5 µM), nifedipine (10 µM) and verapamil (10 µM) (n = 5 for each case), respectively. * showed P<0.05 in comparison with [Ca2+]o = 10 mM group. (TIF) [file pone.0107217.s001.tif]

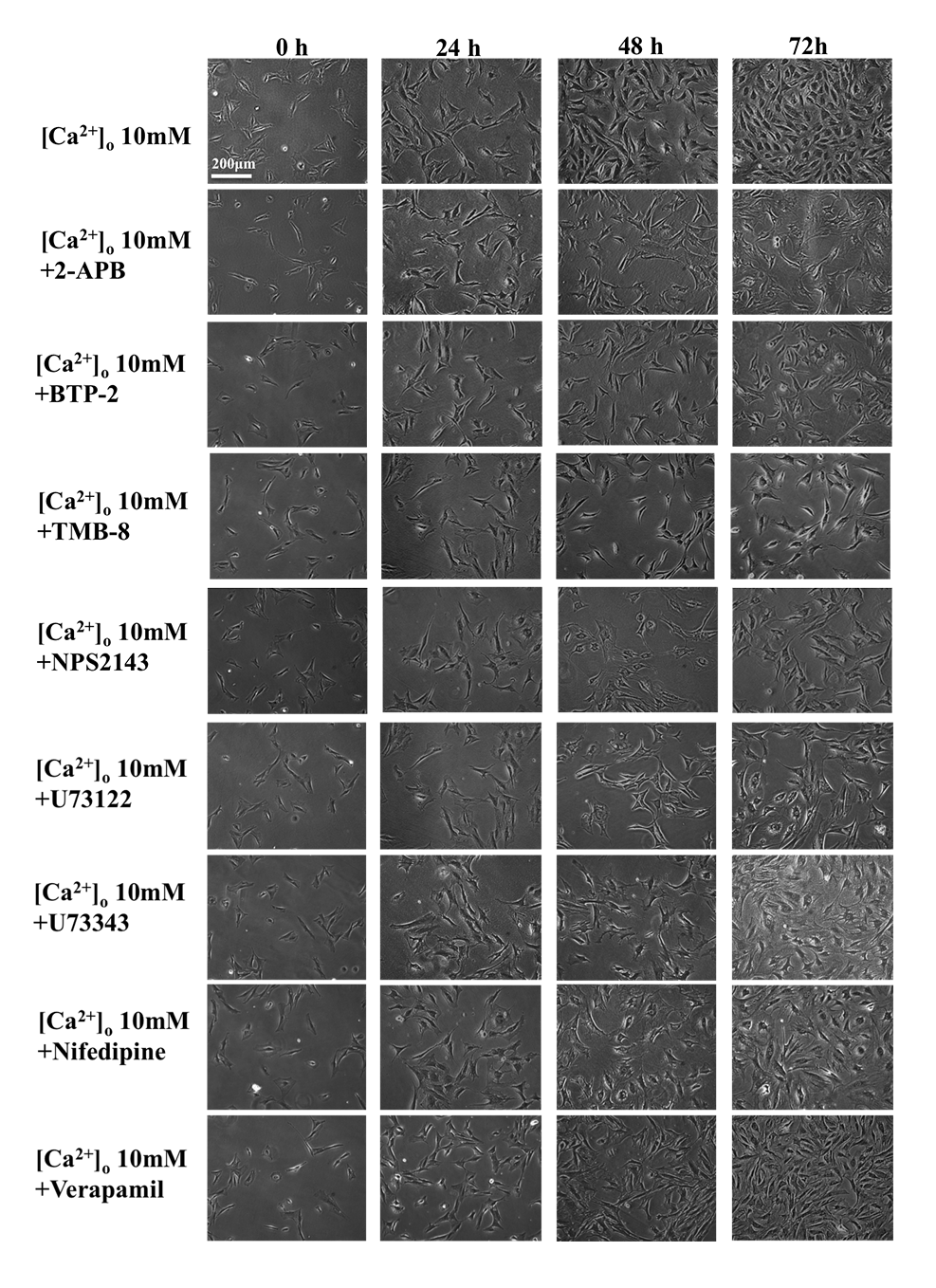

Supplement: Figure S2 — High [Ca2+]o-induced increase in cell numbers was blocked by inhibitors including 2-APB, BTP-2, TMB-8, NPS2143 and U73122, respectively, but not affected by voltage-gated calcium channels blockers nifedipine and verapamil in rat calvarial osteoblasts. Osteoblasts were cultured in medium with 10 mM [Ca2+]o alone or together with 2-APB (25 µM), BTP-2 (20 µM), TMB-8 (50 µM), NPS2143 (10 µM), U73122 (5 µM), U73343 (5 µM), nifedipine (10 µM) and verapamil (10 µM). Representative cell morphological images were captured at 0 h, 24 h, 48 h and 72 h using a 10× objective. (TIF) [file pone.0107217.s002.tif]
